# Supplementary material for: Effect of intravitreal bevacizumab for retinopathy of prematurity on weight gain
Source: PLoS One. 2021 Dec 10;16(12):e0261095. doi: 10.1371/journal.pone.0261095 (PMC8664189; doi:10.1371/journal.pone.0261095)
Supplement: Attachment — Submitted filename: 2021019 ROP-BW response to reviewers comments.docx [file pone.0261095.s002.docx]

**Point-by-Point Responses to Reviewers’ Comments**

Re: “Effect of Intravitreal Bevacizumab for Retinopathy of Prematurity on Weight Gain” by Obata et al.

Please note that our revisions in the manuscript file are shown in red font.

Reviewer #1: Well written article. Good research question. It would also be interesting to have data regarding feeding habits during the period post bevacizumab

Was there a reduction in intake?

**Response: Figure 4, page 6, lines 97–99; page 8, lines 148–158; page 11, lines 197–207.** We thank the reviewer for these supportive comments. To examine the physiological changes before and after treatment, we additionally collected data on blood pressure, pulse rate, oxygen concentration set on the ventilator, volume of milk sucked, and urine output. The IVB and PC groups both showed a temporary decrease in the volume of milk sucked after treatment. The decrease was numerically greater in the IVB group compared with the PC group, but without statistical significance. Although we could not exclude the possibility that decreased feeding may have contributed to the poor BW gain, the mechanism by which IVB caused poor BW gain remained unclear.

Reactivation post bevacizumab is usually from 6 weeks to 35weeks as per Beat ROP study. Any specific reason for repeating intravitreal bevacizumab at 4 weeks

**Response:** At our institution, we do not re-administer bevacizumab at 4 weeks.

Reviewer #2: Since the basis of poor weight gain immediately after bevacizumab is attributed to some systemic effect which could be neurological and hormonal, were there any other side effects like changes in oxygen demands, urine output, blood pressure fluctuations, seizural activity or ever heart rhythm changes?

**Response: Figure 4; page 10, lines 175–179; page 11, lines 197–207.** We thank the reviewer for the suggestions to improve the quality of our manuscript. To examine the physiological changes before and after treatment, we additionally collected data on blood pressure, pulse rate, oxygen concentration set on the ventilator, volume of milk sucked, and urine output as data that we could collect at our institution. As mentioned in the Discussion section, the required oxygen concentration before treatment was numerically slightly higher in the IVB group, but the difference was not statistically significant. High oxygen concentrations interfere with the growth of retinal blood vessels, which may be related to the higher number of Zone 1 cases in the IVB group. There were no changes in blood pressure, pulse rate, and urine output between before and after treatment in the IVB and PC groups. The required oxygen concentration after treatment in the IVB group was higher than that in the PC group, albeit without statistical significance, but it remained unclear whether the use of high oxygen concentrations had an effect on the poor weight gain and the mechanism for how high oxygen concentrations affected BW gain was also unclear. Both groups showed a temporary decrease in volume of milk sucked after treatment. The decrease was greater in the IVB group compared with the PC group, but without statistical significance. Although we could not exclude the possibility that decreased feeding may have contributed to the poor BW gain, the mechanism by which IVB caused poor BW gain remained unclear.
